# Growth, Ecophysiological Responses, and Leaf Mineral Composition of Lettuce and Curly Endive in Hydroponic and Aquaponic Systems

**DOI:** 10.3390/plants13202852

**Published:** 2024-10-11

**Authors:** Lucia Vanacore, Christophe El-Nakhel, Giuseppe Carlo Modarelli, Youssef Rouphael, Antonio Pannico, Antonio Luca Langellotti, Paolo Masi, Chiara Cirillo, Stefania De Pascale

**Affiliations:** 1 Department of Agricultural Sciences, University of Naples Federico II, 80055 Portici, Italy; lucia.vanacore@unina.it (L.V.); christophe.elnakhel@unina.it (C.E.-N.); youssef.rouphael@unina.it (Y.R.); antonio.pannico@unina.it (A.P.); chiara.cirillo@unina.it (C.C.); depascal@unina.it (S.D.P.); 2 Leibniz Institute of Vegetables and Ornamental Crops (IGZ), 14979 Großbeeren, Germany; 3 Centre for Innovation and Development in the Food Industry (CAISIAL), University of Naples Federico II, Via Università 100, 80055 Portici, Italy; langello@unina.it (A.L.L.); pmasi@unina.it (P.M.)

**Keywords:** *Lactuca sativa* L., *Cichorium endivia* L. var. *crispum*, gas exchange, chlorophyll a fluorescence, nitrate, mineral composition, coupled aquaponic

## Abstract

Against the backdrop of climate change, soil loss, and water scarcity, sustainable food production is a pivotal challenge for humanity. As the global population grows and urbanization intensifies, innovative agricultural methods are crucial to meet rising food demand, while mitigating environmental degradation. Hydroponic and aquaponic systems, has emerged as one of these solutions by minimizing land use, reducing water consumption, and enabling year-round crop production in urban areas. This study aimed at assessing the yield, ecophysiological performance, and nutritional content of *Lactuca sativa* L. and *Cichorium endivia* L. var. *crispum* grown in hydroponic and aquaponic floating raft systems, with *Oreochromis niloticus* L. integrated into the aquaponic system. Both species exhibited higher fresh biomass and canopy/root ratios in hydroponics compared to aquaponics. Additionally, hydroponics increased the leaf number in curly endive by 18%. Ecophysiological parameters, such as the leaf net photosynthesis rate, actual yield of PSII, and linear electron transport rate, were also higher in hydroponics for both species. However, the nutritional profiles varied between the two cultivation systems and between the two species. Given that standard fish feed often lacks sufficient potassium levels for optimal plant growth, potassium supplementation could be a viable strategy to enhance plant development in aquaponic systems. In conclusion, although aquaponic systems may demonstrate lower productivity compared to hydroponics, they offer a more sustainable and potentially healthier product with fewer harmful compounds due to the reduced use of synthetic fertilizers, pesticides, and the absence of chemical residue accumulation. However, careful system management and monitoring are crucial to minimize potential contaminants.

## 1. Introduction

In perspective of the problems that must be faced nowadays, such as the increase in the global population that will require up to 50 percent more food production, the increase in greenhouse gas emissions, in addition to the intensification of deforestation, water scarcity, and soil erosion, there is a need to identify new food systems and sustainable farming techniques [1,2]. The COVID-19 pandemic, the economic crisis, and the ongoing wars have underlined the importance of having a more sustainable and resilient food system that can work in all conditions [3,4]. Additional goals to support the sustainability of food and agricultural production systems are promoted by accords established during international protocols and strategies, such as the Milan Urban Food Policy Pact (MUFPP) and Farm-to-Fork (F2F), integral to the Commission’s Agenda for United Nations Sustainable Development Goals (SDGs), aimed at reducing the use of synthetic fertilizers and pesticides [3,5,6,7].

Urban agriculture could represent an efficient tool to mitigate environmental impacts and enhance food security in urban areas [8,9,10]. For this aim, cities could be subjected to the introduction of innovative crop-growing systems that avoid limitations, such as soil degradation and limited space [11,12]. One of the possible options is represented by controlled environment agriculture (CEA) [13,14]. In this initiative, a soilless system is readily integrated into different urban agricultural contexts, including rooftop gardens, community gardens, abandoned greenhouses, and vertical farms [15,16]. Hence, it is considered a viable strategy to address the changing agricultural landscape, providing healthy and high-quality crops and localized year-round production, while reducing the transportation costs associated with traditional soil-based agriculture [17,18]. In addition, these techniques allow natural ecosystems to recover lands that were lost to farming, while also reconnecting citizens with the food they consume, creating new jobs and a more resilient, sustainable, and localized food supply chain [14,18,19,20]. Nowadays, the two most innovative soilless cultivation systems that are spreading in cities around the world are hydroponic (H) and aquaponic (AQ) systems [21].

A hydroponic system is a soilless cultivation technology that applies nutrient solutions and artificial growing media, providing the ability to grow plants in a shorter growing period, in poor soil quality areas, or in limited space all year round, regardless of the climate [22,23]. Despite these advantages, questions remain about the sustainability of this food production system due to its complete dependence on chemical fertilizers. In hydroponics, macro- and micro-nutrients are supplied to plants by dissolving synthetic fertilizers in water to create the nutrient solution [22]. The production of these fertilizers requires significant energy inputs and contributes to increased production and transport costs, as well as higher greenhouse gas emissions [22,24,25].

Conversely, one of the most efficient and environmentally friendly growing techniques is the aquaponic system [26,27,28], a soilless growing technology that combines the production of aquatic organisms and plants in the same environment. The main approaches include either a single recirculating water loop, also known as a coupled system, or a double recirculation system characterized by a physical separation between the aquaculture and hydroponic component, known as a decoupled system [29,30]. In aquaponics, due to the action of two different bacteria groups (i.e., *Nitrosomonas* spp. and *Nitrobacter* spp.), nutrients from fish waste (especially nitrogen compounds) are converted and used by plants into one or more loops [31]. Thusly, nitrification and plant biofiltration reduce the need for complete water exchange, the addition of chemical fertilizers (averagely −50%), and the accumulation of compounds such as ammonia that is potentially toxic to fish [31,32,33,34].

Leafy vegetables are the most grown crops in aquaponics due to their short growing period and better profitability [35,36]. Furthermore, they grow well in water with a high concentration of nitrogen and have low nutrient requirements [35]. Lettuce (*Lactuca sativa* L.) is one of the most cultivated crops hydroponically worldwide [37], and approximately 68% of commercial aquaponics farmers grow lettuce [38]. Regarding the genus *Cichorium*, it is widely known for its antioxidant and anti-inflammatory capabilities related to the presence of several specialized metabolites, including unsaturated fatty acids, alkaloids, flavonoids, saponins, and tannins. *Cichorium endivia* L., the species belonging to this genus, is one of the most consumed salads in the world [39]. In the Mediterranean area, endives are cultivated either in open fields or under high plastic tunnels or multi-span plastic greenhouses [40]. With a production of 27,895 tons, ‘’Campania’’ is the first producer in Italy, and this cultivation represents an important economic activity for this region [41].

Previous literature has primarily focused on the performance of lettuce in hydroponics and aquaponics by investigating several aspects, such as the effects of pH, the application of different nutrient solutions, and the use of different growing methodologies [42,43,44,45]. There are only a few studies on curly endive under these growing conditions, particularly in aquaponics [46,47]. On the other hand, several studies have compared the yield and quality of different crops grown in hydroponic and aquaponic systems, including leafy green vegetables such as lettuce and spinach, as well as fresh herbs including basil, parsley, and rocket along with fruiting crops such as tomatoes [12,48,49,50,51,52]. Other studies have evaluated several aspects related to growth, metabolism, and quality of endive cultivated under the hydroponic system [53,54,55,56]. Moreover, other authors have compared the growth performances, physiological responses, and quality of lettuce and curly endive grown in a recirculating aquaponic system prototype under different lighting treatments [46,47].

However, no research seems to compare endive performance in hydroponic and aquaponic conditions. Therefore, a deeper study is required to understand thoroughly the potential benefits of a coupled aquaponic system. The current study aimed to compare hydroponic and coupled aquaponic floating raft systems for the production of lettuce (*Lactuca sativa* L.) and curly endive (*Cichorium endivia* L. var. *crispum*), with a major focus on yield, ecophysiological behaviour, and some nutritional aspects.

## 2. Results

The initial two-way ANOVA of species vs. cultivation system revealed widespread interaction for almost all variables examined (Table 1), reflecting the differential capacity of both species in the different cultivation systems. Then, a further Student’s *t*-test was performed within each species with respect to hydroponic and aquaponic floating raft systems.

### 2.1. Plant Growth

Lettuce exhibited a significant 26% more canopy FW in the hydroponic cultivation system compared to the aquaponic system, while curly endive exhibited 125.1% (Table 2). More specifically, the lowest values of canopy FW and total leaf area were recorded in curly endive grown under the aquaponic system; whereas, the highest yield was obtained for both the species grown under hydroponic conditions. In addition, curly endive grown under the hydroponic system showed the highest number of leaves (plant^−1^). Compared to aquaponic plants, hydroponic plants allocated more biomass to the canopy, with the canopy/root ratio being significantly higher by 57.4% in curly endive and 45.8% in lettuce compared to aquaponic plants.

### 2.2. Gas Exchanges and Chl “a” Fluorescence Emission

Leaf net photosynthesis (P_n_), the actual yield of PSII (Φ_PSII_), and the liner electron transport rate (ETR) followed the same trend, being lower in lettuce under the aquaponic system and higher in both species grown in hydroponic conditions (Table 3). A comparable pattern was observed for the intrinsic water use efficiency (_i_WUE), which was 3.0-fold and 1.9-fold higher in lettuce and curly endive, respectively, under hydroponic conditions. Additionally, the maximal photochemical efficiency of PSII (F_v_/F_m_) was also higher under the hydroponic system in comparison to the aquaponic one (0.79 and 0.75, respectively, in both species). Lastly, the lowest value of relative water content (RWC) was observed in curly endive grown under the hydroponic system, with aquaponics exhibiting 6.0% higher RWC% compared to the hydroponic cultivation system.

### 2.3. Leaf Photosynthetic Pigments

The content of total chlorophylls and the SPAD index were significantly higher in the hydroponic conditions compared to the aquaponic conditions for both species (Table 4). Specifically, the SPAD index was 101.8% and 128.8% higher in lettuce and curly endive, respectively. In contrast, total carotenoids were 1.1-fold higher in lettuce grown under hydroponic conditions compared to aquaponic conditions, while no significant differences were observed for curly endive.

### 2.4. Mineral Profile and Nitrate Contents

The highest value of nitrate content in leaf tissue was recorded in curly endive grown under the hydroponic system, showing an increase of 55.8% compared to aquaponics. In contrast, the nitrate content in lettuce did not vary statistically among the two cultivation systems. The phosphorus (P) content in lettuce was similar in both cultivation systems, while the lowest value was observed in curly endive grown under hydroponics (Table 5). Concerning the potassium (K) content, it increased by 63.5% in lettuce under hydroponic system, while in curly endive, it did not vary statistically between the two cultivation systems. Calcium (Ca) and magnesium (Mg) concentrations were higher in the aquaponic system than the hydroponic system for both species. Moreover, the highest content of chloride (Cl) was observed in curly endive grown under the aquaponic system; whereas, the lowest one was observed for the same species grown under the hydroponic system. In curly endive, sulphur (S) and sodium (Na) concentrations showed a similar trend, increasing when cultivated under the aquaponic system. In contrast, S did not vary in lettuce between the two cultivation systems, while Na increased by 1.4-fold in the aquaponic one. Finally, the ratio of potassium to sodium increased in both species grown under hydroponics by 2.3-fold and 2.8-fold for curly endive and lettuce, respectively.

## 3. Discussion

In our study, we found that the fresh biomass showed a decreasing trend in the aquaponic system compared to the hydroponic system (Table 2). The aquaponic system significantly decreased the plant growth by reducing the fresh weight in both species and the leaf area in curly endive. Similar results show a reduction in the marketable yield of lettuce and basil in an aquaponic system by 33% and 44%, respectively, in comparison to a hydroponic system, while tomato yields remain comparable between the two cultivation systems [44]. In contrast, studies show that lettuce grown under an aquaponic system during three seasons (winter, spring, and summer), in all cases, equals or improves under the hydroponic equivalent [51]. Further research reports that by supplementing fish water with mineral salts and maintaining a pH of around 5.5, lettuce fresh weight increases by 39% compared to hydroponics [57]. On the other hand, other findings indicate that a pH of 6 is ideal for aquaponic systems to increase the production of six plant species including lettuce without compromising bacterial communities and fish yield [42]. However, in our aquaponic growing condition, the pH of the solution averaged 6.8, which is in compliance with the literature [58,59], while the nutrients came exclusively from fish diet (preformulated fish feed with 42% of protein content); therefore, no addition of micro- and macro-nutrients was performed. Concerning the root fresh biomass, there were no significant variations. Similar results were reported in a study comparing lettuce performance under conventional hydroponics at pH 5.8, hydroponics at pH 7.0, and recirculated aquaponic water at pH 7.0 [60].

From an ecophysiological point of view, hydroponically grown plants showed a higher performance in comparison to aquaponics (Table 3), in line with the yield (Table 2). Our results were likely attributable to the increase in nitrogen content, as suggested by the SPAD (soil plant analysis development) parameter, consistent with research conducted on soybeans [61]. In particular, the F_v_/F_m_ parameter represents the maximal photochemical efficiency of PSII and is used as a sensitive indicator of plants’ photosynthetic performances [62,63]. In our study, F_v_/F_m_ in plants grown under the hydroponic system tended to be higher than the aquaponic one: 0.79 and 0.75, respectively. As a result, the values were close to 0.83, suggesting that the health of hydroponic plants was better [64]. Additionally, the actual yield of the photosystem II (Φ_PSII_) parameter is utilized in photochemistry to determine the proportion of the light absorbed by chlorophyll associated with PSII. Consequently, it can indicate the overall photosynthesis process by measuring the rate of linear electron transport (ETR) [62]. In this study, the increase in F_v_/F_m_ observed in hydroponic plants was in line with the increase in these other physiological parameters as well as with the net photosynthesis rate (P_n_). Comparable physiological trends on different wheat cultivars under heat stress have been observed [65]. However, other research reports conflicting results with ours, finding no significant differences in the photosynthetic parameters of lettuce grown in aquaponic and hydroponic systems [42].

In terms of quality, leafy green vegetables play an important role, as they provide an adequate amount of vitamins and minerals necessary for human health [66]. Lettuce (*Lactuca sativa* L.) and curly endive (*Cichorium endive* L. var. *crispum*) are included among the greatest accumulators of nitrates, and this represents a potential risk to consumer health [67,68], as well as implying a decrease in the product quality [69]. For lettuce cultivated under cover and harvested between April 1 and September 30, the maximum level of nitrates is defined by European Commission Regulation (EU) no. 1258/2011 (http://data.europa.eu/eli/reg/2011/1258/oj, accessed on 15 December 2023) at 4000 mg NO_3_ kg^−1^ fresh weight. Since there is no maximum nitrate level specified for endives, in this study, we took into account the allowable limit for leafy vegetables [70]. In agreement, in our growing conditions, the nitrate concentration was below the limit in both species. Curly endive in particular accumulated more nitrates than lettuce (+2.1-fold); although, the nitrate level in leaf tissue recorded was statistically similar in both the hydroponic and aquaponic systems (Table 5). Our results are consistent with those of other studies [71], which despite recording higher concentrations than the levels accredited by the European Commission, observe no significant difference in nitrate leaf concentration between lettuce grown in a decoupled aquaponic system and a conventional hydroponic control.

Regarding the solubility of essential elements, it is regulated by pH, which can influence the nutrients’ bioavailability for plant uptake [42,72]. It is also a crucial factor in the aquatic system, since it regulates the activity of bacteria and fish metabolism [73,74,75]. In our growing conditions, with similar pH and EC values between hydroponics and aquaponics, there was a significant increase in phosphorous, chloride, sulphur, and sodium concentrations in curly endive grown in the aquaponic system. In contrast, the amounts recorded in lettuce were comparable between the two cultivation systems, except for sodium, which was higher in aquaponics compared to hydroponics. Moreover, calcium and magnesium concentrations were significantly higher in aquaponics in both species (Table 5). Similar results report that lettuce cultivated under the aquaponic system shows higher calcium concentrations than lettuce grown under the hydroponic one. Conversely, magnesium concentrations are lower in aquaponics than hydroponics, while the concentrations of the remaining macronutrients are comparable between the two cultivation systems [43]. Additional studies also report higher values of magnesium in four lettuce varieties grown in hydroponics compared to aquaponics, while phosphorus concentrations show no trend, which is consistent with our results on lettuce [76]. Nevertheless, phosphorus concentrations in the referenced study are lower than the values we recorded in our study (5.55 g kg^−1^ DW on average) [76]. Likely, the higher amounts in fish water allowed our aquaponic plants to accumulate more than hydroponic plants. As regards potassium concentrations, several studies indicate that this element is more deficient within the aquaponic system compared to other elements, resulting in a reduction in plant growth in comparison to the hydroponic system. This deficiency can be attributed to the fact that fish need less potassium in their diet (1% of the composition) [36,75,77,78,79]. In agreement, in our study, we found that potassium was significantly lower in aquaponic- than hydroponic-grown plants, specifically in lettuce. However, potassium is not the only element deficient in aquaponics; fish have minimal requirements for many metal ions, such as iron (Fe), manganese (Mn), magnesium (Mg), and copper (Cu) [58,80], which accordingly lack in fish feed but are required by plants. In particular, iron is essential for several processes, including photosynthetic activities [81,82,83]. In our study, where no micro- or macro-nutrients were introduced, the lack of iron could explain the photosynthetic performance and the lower growth observed in the aquaponic system [84]. Hence, a suitable solution to attend to the full potential of an aquaponic system and achieve economic viability involves small nutritional supplementations, in accordance with the specific requirements of plants [31,85,86].

Given the limited body of literature addressing the soilless cultivation of endives, particularly within aquaponic conditions, our results help to demonstrate that curly endive has proven to be a promising plant for aquaponic cultivation.

## 4. Materials and Methods

### 4.1. Facility

The trial was conducted in a greenhouse (40°48′57.9′′ N 14°21′01.6′′ E, 29 m a.s.l.) in the Department of Agricultural Sciences of the University of Naples Federico II (Portici, Italy). It lasted 23 days, from 27 April to 20 May 2021, and involved both a floating hydroponic raft system and a recirculating aquaponic system (RAS) prototype. Four 2800 L rearing tanks with tilapia fish (*Oreochromis niloticus* L.) in four different weight classes were part of the RAS. The fish were raised in these tanks at a mean stocking density of 8.7 ± 5.4 kg m^−3^. The system consisted of a 400 L trickling filter (Scubla srl, Udine, Italy), a 40 W UV sterilization unit, and an 800-L Superbead system for mechanical and biological filtration (Air-aqua, Wethouder Ohmannstraat, Staphorst, The Netherlands). The tanks’ ambient air insufflation was set at 0.05 v v^−1^ min ^−1^. In the aquaponic treatment (AQ), the floating raft units were connected to the RAS by a single loop, while in the hydroponic treatment (H), it was disconnected and monitored separately.

### 4.2. Planting and Growth Conditions

Plant materials consisted of two-week-old seedlings of lettuce (L), *Lactuca sativa* L. var. “Meraviglia d’inverno”, and curly endive (CE), *Chicorium endivia* var. *crispum* “De Louvriers” (Seedsselect) raised in polystyrene sowing trays. After removing the roots from the peat cube using tap water, they were planted at a density of 20 plants per m^−2^ into the floating raft system of the RAS and hydroponic units, which have a respective area of 2 m^2^. In each system, the water temperature was set to 25 °C, and the average pH and electrical conductivity (EC) were 6.8 and 753 µS cm^−1^, respectively.

### 4.3. Nutrients Solution Management

The RAS unit was supplied with preformulated fish feed containing 42% protein content (Tilapia Grower 13-EF, Alltech Coopens, Helmond, The Netherlands). Fish weight and stocking were used to determine the daily fish feed target; during the trial, the average daily feed consumption was 1.4 ± 0.5% body weight.

For the hydroponic treatment, a half-strength Hoagland nutrient solution made with osmotic water was used. Every two weeks, the solution was reintegrated, and nitric acid (HNO_3_) or potassium hydroxide (KOH) was used to adjust the pH. Daily monitoring of water temperature, pH, and EC was performed for each system (Thermo Scientific Expert pH and Cond Testers, Segrate, Italy). The mineral concentration of both nutrient solutions is listed in Table 6.

### 4.4. Plant Growth and Leaf Traits

Twenty-three days after planting (DAP), a final harvest was performed on 18 plants species^−1^ cultivation system^−1^. Plants’ canopy and roots were separated and weighed using an electronic balance in order to obtain the fresh weights (FW); whereas, the dry weights (DW) were obtained after oven-drying the samples at 70 °C for 48 h. The canopy/root ratio was determined by the ratio of the DW of the root and the DW of the canopy. The number of leaves was also recorded. Afterwards, the counted leaves were utilized for assessing the total leaf area by means of digital images of the leaf lamina using image analysis software (ImageJ software, version 1.50i, Wayne Rasband National Institute of Health, Bethesda, USA). The relative water content (RWC) was determined considering the fresh weight, turgid weight (after overnight with distilled water), and dry weight of 3 fully expanded leaves per replicate.

### 4.5. Gas Exchanges and Chl “a” Fluorescence Emission Determination

Gas exchange measurements were performed at 22 DAP on 1 fully expanded leaf from each replicate using a photosynthesis yield analyzer (LCi T, ADC Bioscientific Ltd., Hoddesdon, UK). Leaf net photosynthesis rate (P_n_) and stomatal conductance (g_s_) were measured at noon in ambient CO_2_ (434 ppm) at a mean temperature of 31.1 °C, relative humidity of 45%, and an average photosynthetic photon flux density (PPFD) of 1251.8 µmol m^−2^ s^−1^. The parameter intrinsic water use efficiency (_i_WUE) derived from P_n_/g_s_ ratio.

Chlorophyll “*a*” fluorescence emission was assessed on the same leaves using a compact plant stress kit, which included a light-adapted Φ_PSII_ meter, a dark-adapted F_v_/F_m_ meter, and 10 dark-adaptation leaf clips (Opti-Sciences Inc., Hudson, TX, USA). Light-adapted measurements were recorded by applying a saturating pulse of 4286 µmol m^−2^ s^−1^ for 1.1 s to obtain the maximum light-adapted fluorescence (F_m′_) and steady-state fluorescence (F_s_). For dark-adapted measurements, leaves were adapted to darkness for 30 min, and then, measurements were recorded by applying a saturating pulse light of 3429 µmol m^−2^ s^−1^ for 1.0 s to obtain the maximal fluorescence (F_m_) and minimal fluorescence (F_0_) values. The PSII maximum photochemical efficiency (F_v_/F_m_) was determined as F_v_/F_m_ = (F_m_ − F_0_)/F_m_. Following the method of Genty et al. [87], the quantum yield of PSII electron transport (Φ_PSII_) was determined as Φ_PSII_ = (F_m′_ − F_s_)/F_m′_ and, using the equation of Krall and Edwards [88], was used to determine the electron transport rate (ETR).

### 4.6. Leaf Chlorophyll and Carotenoids Content and SPAD Determination

Leaf photosynthetic pigment content was measured on 1 fully expanded leaf from each replicate corresponding to those used for the physiological measurements. Leaf samples were promptly frozen at −20 °C until assessment. A 15 mL tube flask was filled with 0.5 g of leaf tissue ground with 5 mL of acetone (80%). After 15 min of dark incubation at room temperature, the solution was centrifuged for 5 min at 3000× *g*. Using a Hach DR 2000 spectrophotometer (Hach Company, Loveland, CO, USA), pigment content was measured by light absorbance at 663, 647, and 470 nm for chlorophyll *a*, *b*, and total carotenoids, respectively. Following Lichtenthaler et al. [89], total chlorophyll was obtained from the sum of chlorophyll *a* and *b*. A portable chlorophyll meter SPAD-502 (Konica Minolta, Tokyo, Japan) was used to determine the SPAD index.

### 4.7. Mineral Profile Determination

A 250 mg fraction of a ground-milled dry leaf sample, obtained using a laboratory grinding mill (model MF10.1, IKA-Werke GmbH & Co. KG, Staufen, Germany), was taken to assess the leaf mineral profile in accordance with Pannico et al.’s [90] methodology. Following a 0.45 µm filter, the concentration of minerals was quantified using an electrical conductivity detector equipped with IonPac AS11-HC and CS12A analytical columns for the analysis of cationic and anionic contents, respectively (Dionex, Sunnyvale, CA, USA). An anion chromatographer (model ICS-3000, Dionex, Sunnyvale, CA, USA) was then used to perform the mineral analysis. All the minerals’ leaf concentrations (P, K, Ca, Mg, Cl, S, and Na) are expressed as g kg^−1^ on a dry weight (DW) basis, with the exception of nitrate, which was reported on a fresh weight (FW) basis based on the dry matter content of the leaves.

### 4.8. Statistical Analysis

Data were initially subjected to a two-way analysis of variance (ANOVA) and interactions were addressed through Tukey’s HSD test. All the data were presented as mean ± standard error, n = 3. Student’s *t*-test, at *p* = 0.05, was performed within each species with respect to the cultivation systems (hydroponic and aquaponic floating raft systems). IBM SPSS Statistics version 29.0.1.0 (SPSS Inc., Chicago, IL, USA) was used.

## 5. Conclusions

This study provides novel insights into the performance of curly endive in an aquaponic system, demonstrating its suitability for this cultivation method. Both hydroponic and aquaponic systems present valuable approaches for producing lettuce and curly endive within controlled environment agriculture (CEA). While aquaponic systems may exhibit slightly lower production rates compared to hydroponic systems, they offer a highly recommended alternative for cultivating healthier produce. Aquaponically grown crops have the potential to be free of chemical contaminants as for pesticide residues, contributing to a more sustainable food sector. However, careful management practices, including the sourcing of inputs and water quality, are crucial to ensuring the absence of contaminants. It is worth mentioning that testing both species in a decoupled aquaponic system could be useful for further increasing the yield and the overall performance of these leafy vegetables.

## Figures and Tables

**Table 1:** Two-way analysis of variance (ANOVA) of all the analyzed variables of both species grown in two different cultivation systems.

Variables	Source of Variance		
	Species (S)	Cultivation System (C)	S × C
Canopy FW	ns	***	***
Number of leaves	**	ns	ns
Total leaf area	ns	**	ns
C/R ratio	ns	***	ns
Roots FW	***	ns	*
P_n_	ns	***	ns
_i_WUE	ns	***	*
F_v_/F_m_	ns	***	ns
Φ_PSII_	*	**	ns
ETR	ns	***	*
RWC	ns	**	ns
Total chlorophylls	ns	*	ns
Total carotenoids	*	ns	ns
SPAD index	ns	***	ns
NO_3_	***	ns	***
P	ns	ns	***
K	ns	***	*
Ca	*	*	ns
Mg	ns	***	ns
Cl	ns	*	***
S	*	ns	***
Na	*	ns	*
K/Na	*	*	*

ns, *, **, and *** mean no significant and significant effects at *p* < 0.05, 0.01, and 0.001, respectively.

**Table 2:** Plant growth in terms of canopy fresh weight (FW), number of leaves, total leaf area, specific leaf area (SLA), canopy/root ratio (C/R), root fresh weight (FW) in lettuce (L) and curly endive (CE) grown under aquaponic (AQ) and hydroponic (H) systems.

Species	Cultivation System	Canopy FW (g FW Plant ^−1^)	Leaf Number (n Plant ^−1^)	Total Leaf Area (cm^2^ Plant^−1^)	C/R Ratio	Roots FW (g FW Plant^−1^)
Lettuce	Aquaponics	199.17 ± 9.04 b	35.33 ± 1.17	3183.87 ± 51.35	3.74 ± 0.1 b	17.69 ± 0.67
	Hydroponics	252.44 ± 13.42 a	39.67 ± 1.76	3623.14 ± 229.43	6.9 ± 1.03 a	15.71 ± 0.65
		*	ns	ns	*	ns
Curly	Aquaponics	121.18 ± 7.53 b	43.22 ± 2.06 b	2585.62 ± 90.2 b	3.27 ± 0.15 b	21.8 ± 1.29
endive	Hydroponics	272.83 ± 7.41 a	53 ± 2.08 a	3534.92 ± 62.68 a	7.68 ± 0.84 a	23.81 ± 0.59
		***	*	***	**	ns

All data are expressed as mean ± standard error, n = 3. Student’s *t*-test was performed within each species with respect to the cultivation systems. ns, *, **, and *** mean no significant and significant effects at *p* ≤ 0.05, 0.01, and 0.001, respectively. Different letters within columns indicate significant mean differences (*p* = 0.05).

**Table 3:** Gas exchanges and chlorophyll “a” fluorescence emission in terms of leaf net photosynthesis (P_n_), maximal photochemical efficiency of PSII (F_v_/F_m_), actual yield of PSII (Φ_PSII_), linear electron transport rate (ETR), intrinsic water use efficiency (iWUE), and relative water content (RWC) in lettuce (L) and curly endive (CE) grown under aquaponic (AQ) and hydroponic (H) systems.

Species	Cultivation System	P_n_ (mol CO_2_ m^−2^ s^−1^)	_i_WUE (µmol CO_2_ m^−2^ s^−1^/mol H_2_O m^−2^ s^−1^)	F_v_/F_m_	Φ_PSII_	ETR (µmol m^−2^ s^−1^)	RWC (%)
Lettuce	Aquaponics	4.81 ± 0.83 b	19.4 ± 3.07 b	0.75 ± 0.01 b	0.23 ± 0.01 b	53.53 ± 6.13 b	93.34 ± 0.87
	Hydroponics	10.49 ± 0.83 a	57.71 ± 5.9 a	0.79 ± 0.01 a	0.36 ± 0.02 a	123.17 ± 10.01 a	90.34 ± 0.8
		**	**	*	**	**	ns
Curly	Aquaponics	5.93 ± 0.65 b	22.53 ± 2 b	0.75 ± 0.01 b	0.34 ± 0.03 b	89.58 ± 9.71 b	91.29 ± 0.73 a
endive	Hydroponics	9.34 ± 0.54 a	41.91 ± 1.83 a	0.79 ± 0.01 a	0.47 ± 0.01 a	118.83 ± 3.23 a	86.14 ± 0.75 b
		*	**	*	*	*	**

All data are expressed as mean ± standard error, n = 3. Student’s *t*-test was performed within each species with respect to the cultivation systems. ns, *, and ** mean no significant and significant effects at *p* ≤ 0.05 and 0.01, respectively. Different letters within columns indicate significant mean differences (*p* = 0.05).

**Table 4:** Leaf photosynthetic pigments content in terms of total chlorophylls (a + b) content, total carotenoids content, and SPAD index in lettuce (L) and curly endive (CE) grown under aquaponic (AQ) and hydroponic (H) systems.

Species	Cultivation System	Total Chlorophylls (mg g^−1^ FW)	Total Carotenoids (mg g^−1^ FW)	SPAD Index
Lettuce	Aquaponics	1.15 ± 0.05 b	0.26 ± 0.01 b	16.34 ± 0.82 b
	Hydroponics	1.49 ± 0.08 a	0.29 ± 0.01 a	32.97 ± 1.34 a
		*	*	***
Curly	Aquaponics	1.07 ± 0.11 b	0.25 ± 0.01	14.51 ± 0.68 b
endive	Hydroponics	1.84 ± 0.13 a	0.24 ± 0.02	33.2 ± 1.42 a
		*	ns	***

All data are expressed as mean ± standard error, n = 3. Student’s *t*-test was performed within each species with respect to the cultivation systems. ns, * and *** mean no significant and significant effects at *p* ≤ 0.05 and 0.001, respectively. Different letters within columns indicate significant mean differences (*p* = 0.05).

**Table 5:** Leaf mineral concentrations, expressed as mg kg^−1^ fresh weight (FW) for nitrate and as g kg^−1^ dry weight (DW) for all others (P, K, Ca, Mg, Cl, S, and Na) in lettuce (L) and curly endive (CE) grown under aquaponic (AQ) and hydroponic (H) systems.

Species	Cultivation System	NO_3_	P	K	Ca	Mg	Cl	S	Na	K/Na
Lettuce	Aquaponics	1456.51 ± 81.34	5.12 ± 0.31	61.49 ± 3.57 b	10.61 ± 0.7 a	3.92 ± 0.35	9.74 ± 0.9	0.97 ± 0.09	1.32 ± 0.09 a	46.88 ± 2.76 b
	Hydroponics	1433.43 ± 65.76	5.99 ± 0.13	100.53 ± 0.66 a	7.69 ± 0.46 b	2.46 ± 0.45	11.08 ± 0.23	1.19 ± 0.02	0.96 ± 0.08 b	105.53 ± 8.5 a
		ns	ns	***	*	ns	ns	ns	*	**
Curly	Aquaponics	2445.27 ± 157.07 b	5.41 ± 0.2 a	82.3 ± 5.88	7.35 ± 0.75 a	3.2 ± 0.21 a	21.68 ± 0.76 a	3.63 ± 0.23 a	4.82 ± 0.8 a	17.69 ± 1.82 b
endive	Hydroponics	3809.69 ± 268.75 a	4.17 ± 0.19 b	97.83 ± 3.3	4.04 ± 0.32 b	1.59 ± 0.13 b	5.53 ± 0.5 b	1.21 ± 0.1 b	2 ± 0.07 b	49 ± 0.42 a
		*	*	ns	*	**	***	***	*	***

All data are expressed as mean ± standard error, n = 3. Student’s *t*-test was performed within each species with respect to the cultivation systems. ns, *, **, and *** mean no significant and significant effects at *p* ≤ 0.05, 0.01, and 0.001, respectively. Different letters within columns indicate significant mean differences (*p* = 0.05). NO_3_: mg kg^−1^ FW and the rest in mg kg^−1^ DW.

**Table 6:** Concentration of nitrate (NO_3_), nitrogen dioxide (NO_2_), phosphorus (P), potassium (K), calcium (Ca), magnesium (Mg), chlorine (Cl), sulphur (S), and sodium (Na) in the hydroponic (H) and aquaponic (AQ) systems.

Cultivation System	Concentration (mg L^−1^)								
	NO_3_	NO_2_	P	K	Ca	Mg	Cl	S	Na
H	218.5	0.14	2.9	26.79	55.5	16.22	2.53	13.65	9.88
AQ	336.49	0.21	7.75	19.55	77.09	20.11	23.72	5.78	26.38

## Data Availability

The data are contained within the article.
